# High Resolution, Large Deformation 3D Traction Force Microscopy

**DOI:** 10.1371/journal.pone.0090976

**Published:** 2014-04-16

**Authors:** Jennet Toyjanova, Eyal Bar-Kochba, Cristina López-Fagundo, Jonathan Reichner, Diane Hoffman-Kim, Christian Franck

**Affiliations:** 1 School of Engineering, Brown University, Providence, Rhode Island, United States of America; 2 Department of Molecular Pharmacology, Physiology and Biotechnology, Center of Biomedical Engineering, Brown University, Providence, Rhode Island, United States of America; 3 Department of Surgery, Rhode Island Hospital, Providence, Rhode Island, United States of America; 4 The Warren Alpert Medical School of Brown University, Providence, Rhode Island, United States of America; 5 Brown Institute for Brain Science, Providence, Rhode Island, United States of America; Tufts University, United States of America

## Abstract

Traction Force Microscopy (TFM) is a powerful approach for quantifying cell-material interactions that over the last two decades has contributed significantly to our understanding of cellular mechanosensing and mechanotransduction. In addition, recent advances in three-dimensional (3D) imaging and traction force analysis (3D TFM) have highlighted the significance of the third dimension in influencing various cellular processes. Yet irrespective of dimensionality, almost all TFM approaches have relied on a linear elastic theory framework to calculate cell surface tractions. Here we present a new high resolution 3D TFM algorithm which utilizes a large deformation formulation to quantify cellular displacement fields with unprecedented resolution. The results feature some of the first experimental evidence that cells are indeed capable of exerting large material deformations, which require the formulation of a new theoretical TFM framework to accurately calculate the traction forces. Based on our previous 3D TFM technique, we reformulate our approach to accurately account for large material deformation and quantitatively contrast and compare both linear and large deformation frameworks as a function of the applied cell deformation. Particular attention is paid in estimating the accuracy penalty associated with utilizing a traditional linear elastic approach in the presence of large deformation gradients.

## Introduction

Traction Force Microscopy (TFM) is a powerful methodology of quantifying cellular forces during cell-material interactions. From the early work developed by Harris et al. in 1980 to the pioneering work by Oliver et al. and Dembo et al. in the mid 1990s, TFM studies have demonstrated the important role that mechanical cues play in cell biology by providing rigorous means of quantifying cellular traction forces [1]–[9]. Starting in 2009, Maskarinec et al. and Hur et al. showed the importance of recording all three traction components in gaining a deeper and fuller understanding of how cells interact with their substrate materials during locomotion and spreading [10]–[12]. Recent studies have underscored the importance of measuring the full three-dimensional (3D)traction profiles during cell migration, locomotion and cell-cell interactions [13]–[16].

TFM computes cell-generated surface tractions from a set of measured cell-induced displacement fields that are typically recorded by using either a single particle tracking or image correlation approach, such as digital image (2D) or digital volume (3D) correlation [2], [6], [9]–[13]. Image correlation techniques are generally advantageous over single particle tracking algorithms if a high enough fiducial tracker particle density can be achieved. They are generally less prone to error in the presence of noise and they can be readily computationally implemented. Once the cell-generated displacement fields are computed, the surface tractions can be calculated either through an inverse Boussinesq formulation or in cases where 3D displacement data is available, in a forward formulation as presented by Franck et al. [11], [12]. Alternatively, one can utilize a finite element framework to compute surface tractions from the measured displacement values [10], [17].

In almost all of the currently reported TFM methods, the underlying assumption is that the cell-generated material strains are small enough to be analyzed within a linear elastic continuum framework [18], [19]. This assumption seems justified for most published work showing mostly small cellular strains.

Utilizing a recently developed advanced high resolution digital volume correlation (DVC) technique [20], we show that cells exert large, or finite, deformations with strain magnitudes of up to 40%. Motivated by these observations, we present a new reformulated approach to calculating cellular traction fields in 3D, using a large deformation formulation. This new methodology is capable of providing unprecedented spatial detail of 3D cellular traction forces with a four to five fold signal-to-noise improvement over our previous small deformations 3D TFM approach [11], [12]. At the same time we reduce the overall computation times by one order of magnitude by implementing our algorithm on a personal computer's graphics processing unit (GPU) [20]. Finally, we quantitatively compare and contrast the differences in calculated cell tractions between the traditional small deformation (SD) (linear) TFM framework and our new large deformation (LD) (non-linear) methodology. We show that for small strains, the LD approach automatically simplifies to the commonly used linear framework, thus providing the most general and robust overall approach. Our entire 3D TFM large deformation algorithm is freely available from our website (franck.engin.brown.edu).

## Results

The large deformation 3D TFM (LD 3D TFM) methodology presented in this paper consists of two basic components, analogous to our previous small deformation 3D TFM (SD 3D TFM) technique. First, a novel fast iterative digital volume correlation algorithm (FIDVC) [20] is used to compute the 3D cell-generated displacement fields from a series of laser scanning confocal microscopy (LSCM) 3D images. Second, the measured 3D displacement fields are converted to cellular tractions using a LD continuum mechanics formulation.

### High Resolution Improvements in Measuring 3D Cellular Displacement Fields

**Figure 1:**
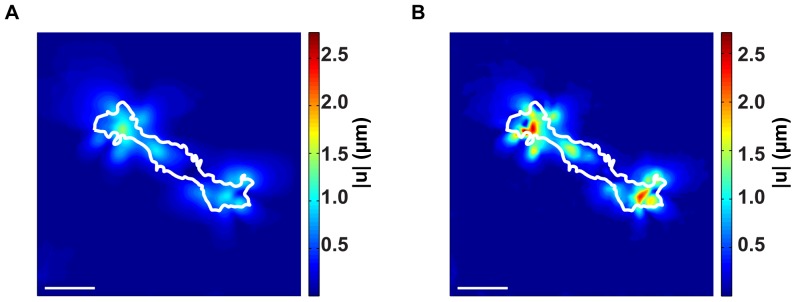
Top-view displacement contours of a migrating Schwann cell measured by DVC and FIDVC. Side by side comparison of the 3D cell displacements measured with (A) our previous DVC [21] and (B) our new FIDVC algorithm [20]. Cell outlines are shown in white. Scale bars = 40 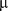
m.

Our newly developed FIDVC algorithm is capable of producing spatial resolutions and accuracies far beyond most current 3D methods, and is a significant advancement over our previously reported DVC displacement algorithm. Figure 1 depicts the cell-induced 3D displacement fields of a Schwann cell during locomotion. For comparison purposes, Fig. 1(A) presents the cell displacement analyzed with our previous DVC algorithm [21], whereas Fig. 1(B) shows the results analyzed with our high resolution FIDVC technique [20]. As can be seen from Fig. 1, the recovered displacement magnitudes as well as their spatial gradients are significantly better resolved using the FIDVC, providing higher overall signal-to-noise ratio and finer spatial detail of the cell-induced material displacement fields than our previous methodology.

**Figure 2:**
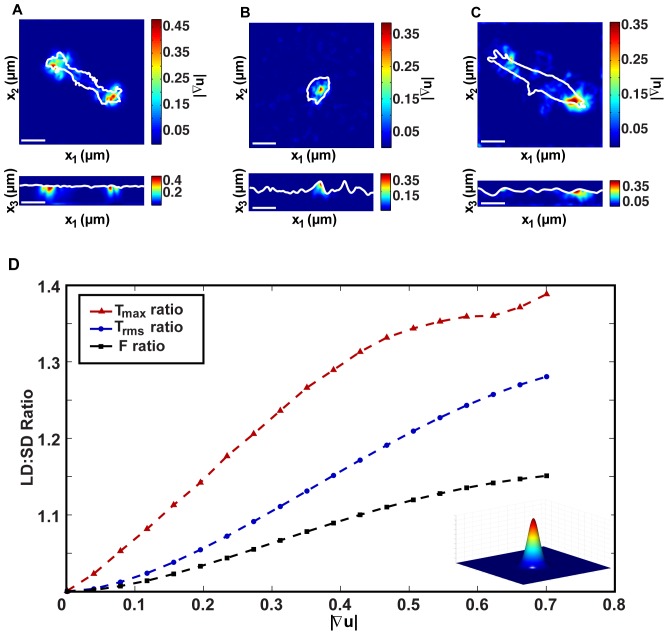
Displacement gradient comparison for large deformation. 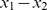
 and 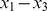 cross-sections of the calculated 3D displacement gradient for (A) a Schwann cell (scale bar = 40 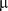
m), (B) a polymorphonuclear leukocyte (scale bar = 20 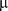
m) and (C) a NIH 3T3 fibroblast (scale bar = 20 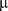
m). (D) Total force (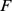), root mean squared tractions (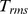) and maximum traction (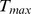) ratios plotted against the displacement gradient, under the application of a 3D Gaussian-shaped displacement field (inset). The numerator in the ratios is calculated using the new large deformation approach, whereas the denominator features the results from the traditional linear elastic, small deformation material approximation.

Due to the improvement in spatial resolution, we found that cells are indeed capable of producing large deformations. Figure 2 presents color contour plots of three different cells seeded on polyacrylamide gels: (A) a Schwann cell, (B) a polymorphonuclear neutrophil, and (C) an NIH 3T3 fibroblast all generating large displacement gradients, or material strains. The choice of plotting the magnitude of the displacement gradient rather than a particular strain component is to show with specific numbers that the non-linear component can no longer be neglected, and that the finite deformation behavior of the material and its associated Lagrangian strains (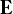) need to be accounted for in Eq. 5. This is further illustrated in Fig. 2(D) showing significant deviations in the traction estimates between the small strain, linear and finite deformation theory as the displacement gradient term increases. Figure 2(D) quantifies the deviation in surface tractions and total force between the LD and SD analyses as a function of the displacement gradient magnitude. This is not to say that linear theory is always inappropriate, but rather to point out that for high cellular displacement gradients the use of small strain theory can produce significant error in reporting accurate cell tractions. We believe these observations are primarily the result of our improved spatial accuracy in better resolving the cell displacement fields. Many of the currently available 3D displacement motion estimation algorithms intrinsically neglect to account for LD in their formulations, and as a result underpredict both the magnitude and gradients of the cell-induced displacement fields [20].

**Figure 3:**
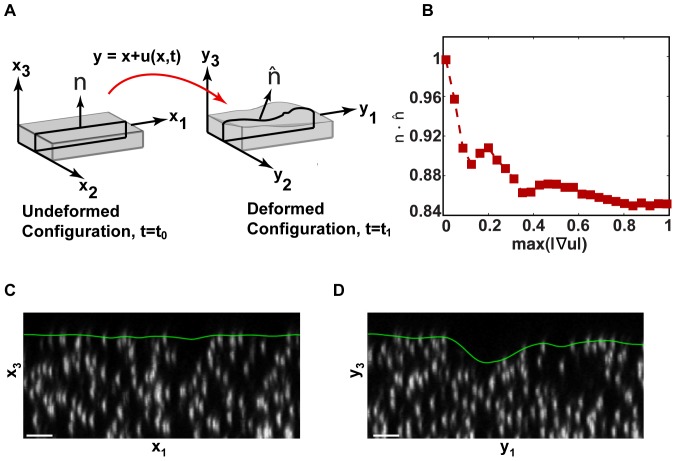
Undeformed and deformed surfaces due to a large deformation. (A) Schematic of how a material deforms from a reference configuration, 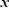, at time 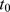, into a deformed configuration, 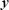, at time 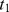. (B) Angle change between the undeformed and deformed surface normals in (A) 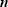 and 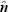, under the application of a cell-simulated Gaussian displacement field profile. The x-axis denotes the maximum value of the full-field 3D displacement gradient magnitude. The dot product represents the cosine of the angle between the two surface vectors. LSCM 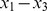 cross-sectional images (C) in the absence of a cell, and (D) directly underneath a locomoting Schwann cell. Scale bars = 5 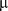
m.

This deviation is illustrated in more detail in Fig. 3. Contrary to small deformation theory that intrinsically assumes that any deformed configuration is the same as the undeformed reference configuration, finite deformation theory features large topographical surface changes as those experimentally observed in Fig. 2(A)–(C). Fig. 3(A) schematically illustrates how the local 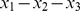 coordinate system of the undeformed (reference) configuration maps into a deformed configuration 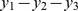 under the application of a large deformation. There are two important conclusions to notice: First, the coordinate system in the deformed state is no longer equal to the coordinate system in the undeformed state, i.e., 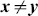. Second, whereas in the undeformed state the 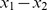 traction components are planar (2D), in the deformed configuration the 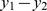 traction components are fully 3D with respect to the original 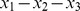 coordinate system. Another way to visualize this is by looking at the angle change between the surface normals of the deformed and reference configuration. This is illustrated in Fig. 3(B). If the reference and deformed surfaces remain the same, as assumed in linear elasticity, then the two normal vectors are identical and their vector dot product, or cosine between the normals, is equal to one. However, as shown in Fig. 3(B), in the presence of a non-negligible displacement gradient, the surface normals can deviate significantly, causing an initially planar surface to deform out-of-plane. Most of the deviation in the 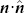 term in Fig. 3(B) is caused by significant material rotations at the free surface. These large surface rotations are seen in Figs. 3(C)–(D) which presents an experimental 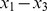 cross-sectional image obtained via confocal microscopy before and after applied cell deformation. The material shown is a polyacrylamide substrate with embedded 0.5 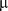
m fluorescent particles, and the green line represents the free surface of the sample. As a result, even if only a 2D representation of the cell-induced tractions is sought, the occurrence of large deformations requires a 3D imaging modality, e.g., confocal microscopy, to resolve those surface shear tractions properly. If instead, only a 2D image is acquired of the embedded fluorescent fiducial markers before and after finite deformation, the recovered displacements can only provide optical projections of the actual 3D material displacements, which can yield significant measurement error.

### Comparison between Large and Small Deformation Traction Results (Analytical Example)

To provide the reader with a more comprehensive analysis of using a small (SD) versus large (LD) deformation framework to calculate tractions, we present two specific examples. In the first example, an analytical, simulated displacement dipole is generated with a displacement gradient magnitude, 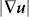 = 0.5, whereas in the second example a displacement field is experimentally measured from a locomoting Schwann cell. In both cases, the surface tractions, forces, and corresponding strain energies are compared using SD and LD frameworks.

To validate our LD 3D TFM methodology and to quantify the differences between a SD (linear) and LD TFM approach, we generate two analytical displacement dipoles applied along a free surface of the 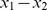 plane, in the form of
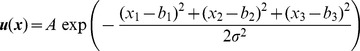
(1)with 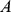 being the amplitude, 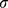 the spread, and 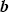 the position of the center peak of the Gaussian function (Fig. 2(D) insert). Here, 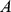 and 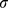 were chosen such that the displacement gradient magnitude, 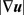, yields a value of 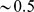, which is similar to the ones experimentally observed (Fig. 2).

**Figure 4:**
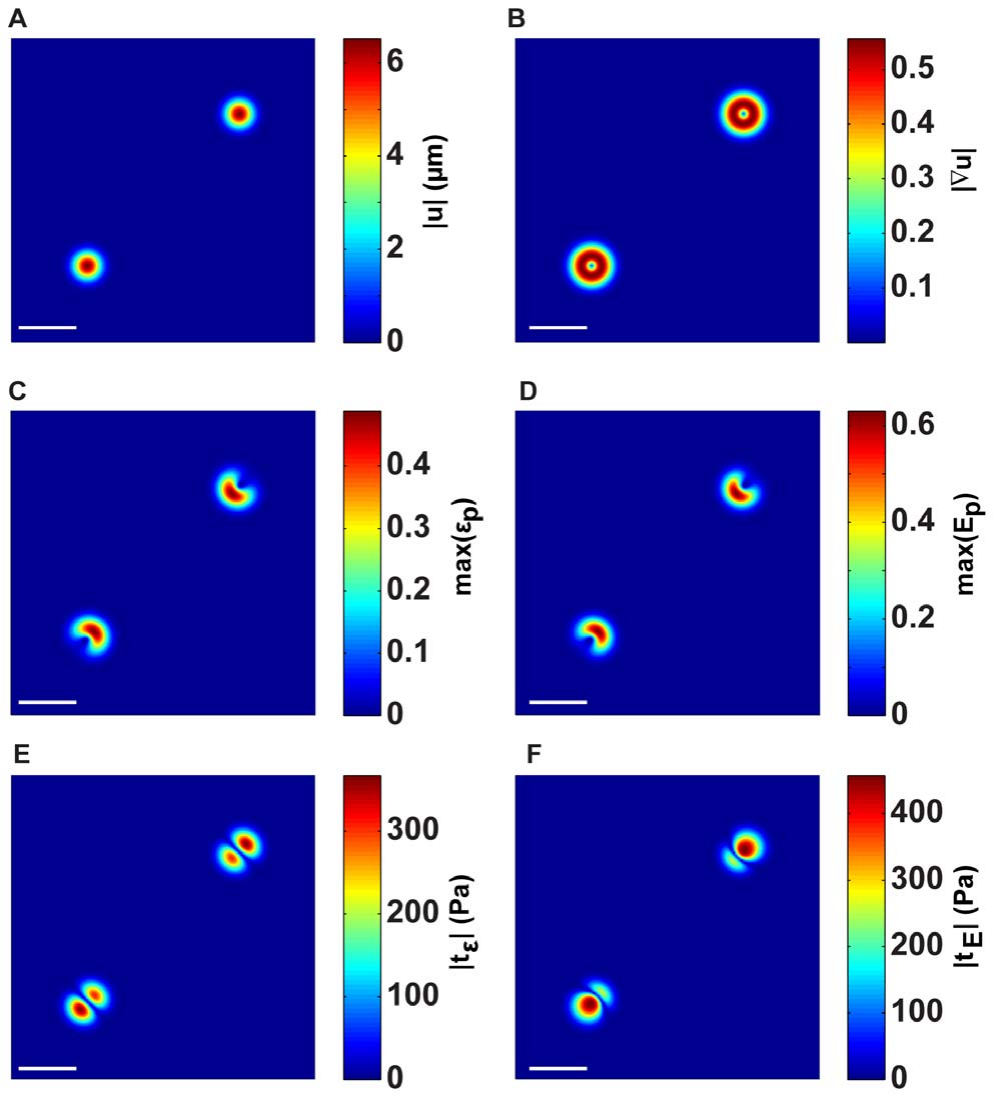
Analytical example of prescribed Gaussian displacement dipoles on the surface of a 3D LSCM imaging volume. The (A) 3D surface displacement magnitude, 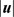, and (B) displacement gradient magnitude, 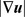. Profiles of calculated maximum 3D principal strains calculated from the (C) infinitesimal (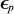) and (D) Lagrangian (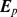) strains. The corresponding traction magnitudes calculated on the (E) undeformed surface, 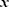, using a linear elastic constitutive model, 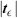, and on (F) the deformed surface, 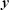 using a large deformation (LD) constitutive model 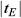. Scale bars = 40 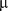
m.

Figure 4(A) plots the magnitude of the 3D displacement vector, 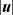, along the 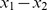 free surface plane, whereas Fig. 4(B) depicts the magnitude of the displacement gradient, 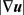 of the analytically applied displacement fields along the same plane. Figs. 4(A)–(B) serve as the input parameters to calculate the surface tractions shown in Figs. 4(C)–(F). For comparison, Fig. 4(C) displays the maximum principal infinitesimal strain (SD), 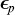, whereas Fig. 4(D) displays the maximum principal Lagrangian strains (LD), 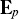. The difference between the two arises from the non-linear displacement gradient term in Eq. 5. The final row in Figs. 4(E)–(F) compares the resulting surface tractions between the SD and LD approaches. Fig. 4(E) presents the surface tractions as calculated by our previous SD 3D TFM methodology, which assumes the material to be linearly elastic, whereas Fig. 4(F) presents the tractions calculated using a finite LD Neo-Hookean material behavior, which has been shown to adequately describe the material behavior of polyacrylamide at large strains [22].

Comparison between Figs. 4(E)–(F) shows that both the magnitude as well as the spatial distribution of the calculated surface tractions are different (see Figure S1). The 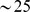
% difference in the recovered traction magnitude is a strong function of the local displacement gradient, as illustrated in Fig. 2(D). The larger the displacement gradient, the stronger the deviation between the two traction fields. The spatial differences in the traction fields are primarily attributed to the underlying topographical change of the substrate surface, which as shown in Fig. 3, changes the spatial distribution of the in-plane and out-of-plane tractions.

**Figure 5:**
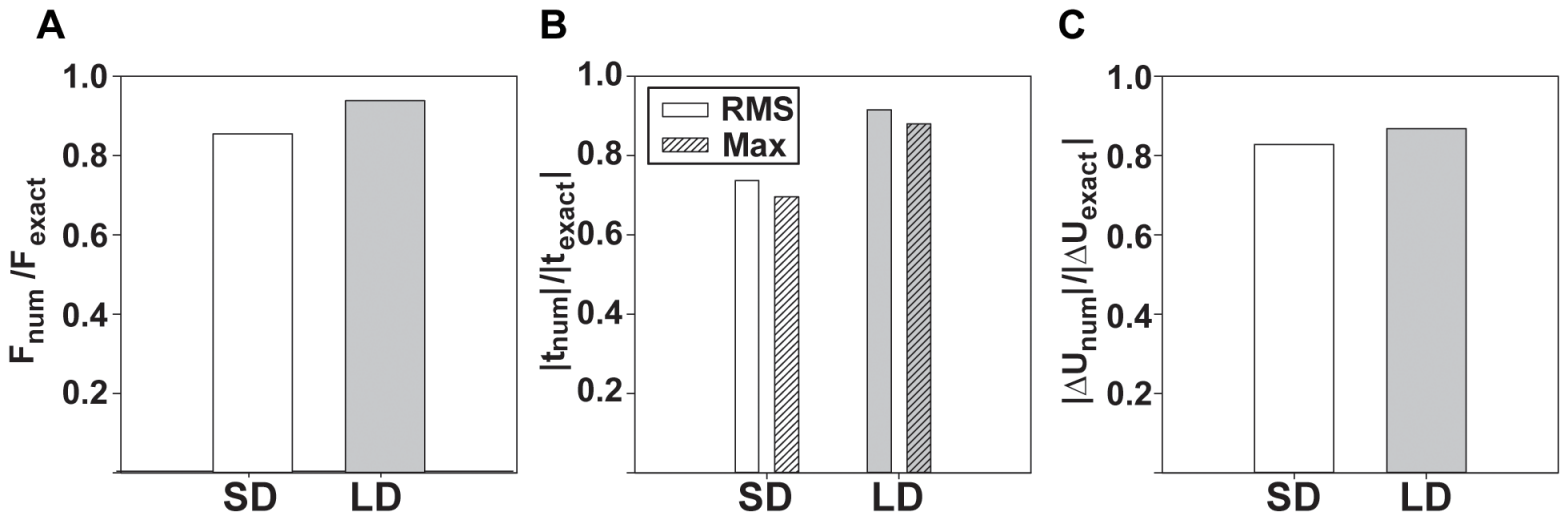
Comparison of commonly reported metrics in TFM for the analytical example. Side by side comparison of the (A) total force, (B) root mean squared (RMS) tractions and maximum tractions, and (C) strain energy for both the linear elastic, small deformation (SD) and non-linear, large deformation (LD) models. All of the values are normalized by the exact analytical solution.

To conclude our validation, we compare the total applied surface forces for both the SD and LD 3D TFM approaches to the analytically computed force. This result is shown in Fig. 5(A), which plots the ratios between the total force magnitudes of the SD and LD 3D TFM approaches to the exact analytical value. As expected, in the presence of high displacement gradients, the linear, small strain (SD) approach underestimates the total force by 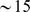
%, whereas our large deformation formulation (LD) predicts it more accurately. The corresponding root mean squared and maximum traction values are compared with the exact solution and presented in Fig. 5(B). Figure 5(C) depicts the ratio in the elastic strain energy ratios between the calculated values and the exact solution. The deviation between the exact solution and the large deformation formulation (LD) is mostly due to numerical errors associated with calculating the material strains from the imposed displacements (see Figure S2).

### Comparison between Large and Small Deformation Traction Results (Experimental Cell Example)

**Figure 6:**
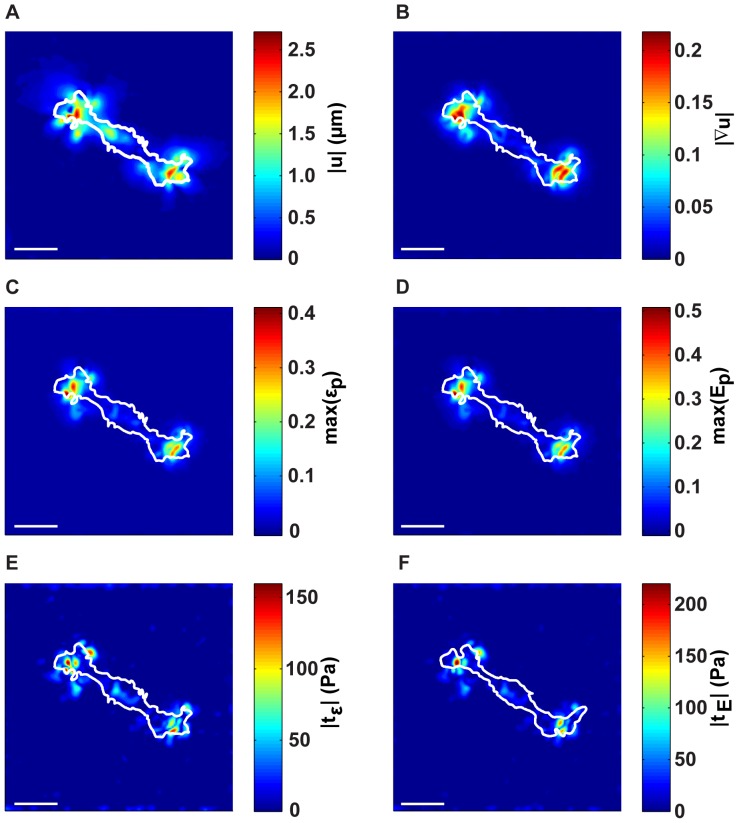
Experimental example of a migrating Schwann cell on the surface of a 3D LSCM imaging volume. (A) Magnitude of the 3D Schwann cell surface displacement field, 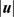, and its (B) resulting displacement gradient magnitude, (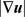). Calculated maximum principal strains from the (C) infinitesimal (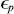), and (D) Lagrangian (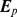) strains. The corresponding traction magnitudes calculated on the (E) undeformed surface, 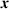, using a linear elastic constitutive model, 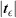, and on the (F) deformed surface, 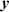 using a large deformation (LD) constitutive model 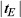. Cell outlines are shown in white. Scale bars = 40 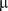
m.

In the experimental cell example, Schwann cells are seeded on laminin-conjugated polyacrylamide substrates as detailed in the Materials and Methods section. To highlight the differences in calculated cellular strain and traction fields, we applied the same comparative approach from the previous section to analyzing the experimental cell displacement data. Figure 6(A) is a plot of the 3D displacement vector field magnitude, 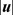, directly underneath a migrating Schwann cell. Fig. 6(B) plots the corresponding displacement gradient magnitude, 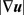, of the measured displacements in Fig. 6(A). Analogous to Fig. 4, Figs. 6(A)–(B) serve as the input data to calculate the cell surface strains and tractions shown in Figs. 6(C)–(F). Figure 6(C) depicts the maximum principal infinitesimal strain, 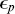, whereas Fig. 6(D) displays the maximum principal Lagrangian strain, 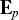. As before, the difference in the strains arises from neglecting the squared gradient term in Eq. 5. Figures 6(E)–(F) plot the respective surface traction magnitudes for the linear elastic method (SD 3D TFM), 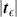, and the finite deformation method (LD 3D TFM), 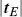. As in Fig. 6(E), the surface tractions are calculated based on our previous SD 3D TFM technique, whereas in Fig. 6(F), the tractions are calculated using our new LD 3D TFM methodology. Similar to our analytical example, the traction magnitude is underpredicted in the linear case. More importantly, the spatial distribution and features of the traction patterns are significantly different between the SD and LD formulations due to differences in the surface topography (illustrated in Fig. 3), and in the constitutive material models (Eq. 9).

**Figure 7:**
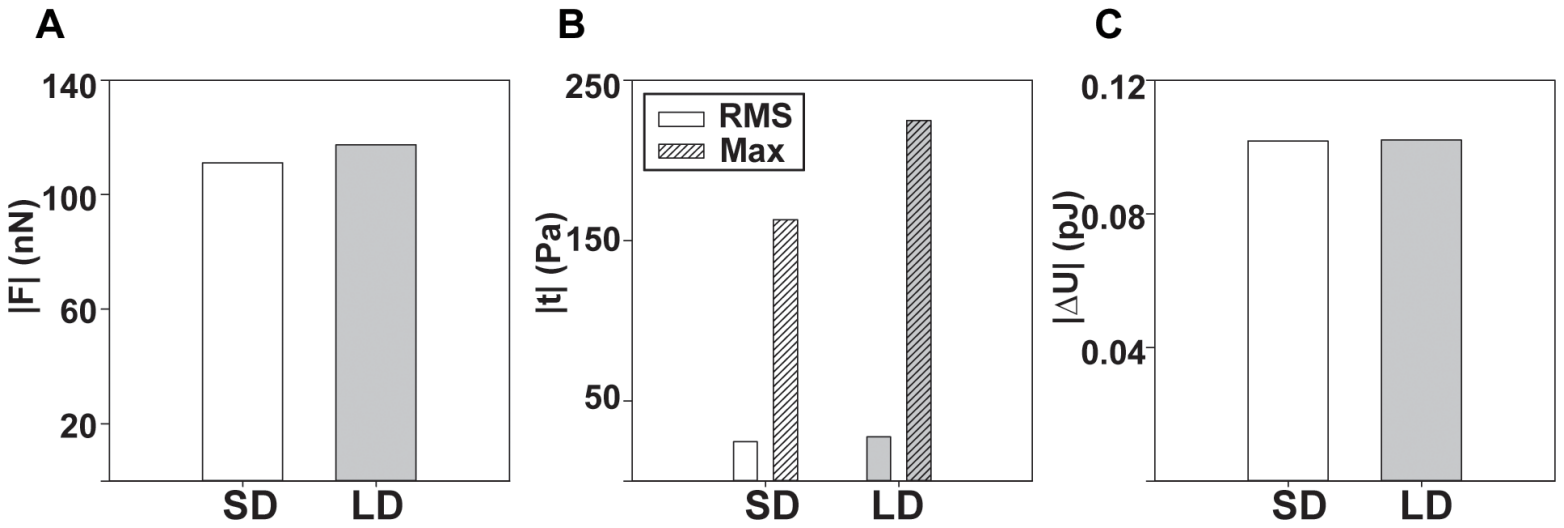
Comparison of commonly reported metrics in TFM for the experimental example. Side by side comparison of the (A) total force, (B) root mean squared (RMS) and maximum tractions, and (C) strain energy for both the linear elastic, small deformation (SD) and non-linear, large deformation (LD) models.

While total force and strain energy are convenient scalar quantities to classify cellular contractility, root-mean-squared and maximum traction measures present other useful metrics that are not dependent on the actual projected cell surface area. Figures 7(A)–(C) highlight the differences in total force, surface tractions, and strain energy between the linear elastic and finite deformation methodologies. When comparing Figs. 7(A)–(C), the deviations between the tractions, in particular between the maximum tractions are much higher than for the forces or strain energies. This is not surprising since the average cell-applied surface tractions generally feature small displacement gradients, as to not produce significant differences between the two models. The maximum tractions, on the other hand, will almost always feature very steep underlying displacement gradients, 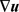, making the calculation extremely sensitive to the deviation in the non-linearity of the particular model used (for more examples, see Figures S3, S4, S5, S6).

## Discussion

This paper presents an advanced high resolution 3D TFM technique capable of calculating fully non-linear cell-induced material stresses and traction fields. By utilizing a recently developed FIDVC technique, cellular displacement fields can be measured with unprecedented resolution and at computation times an order of magnitude faster than our previous 3D DVC algorithm. The increase in computational speed and accuracy is achieved through the extension of the well-known iterative deformation method (IDM) [23]–[25] into three dimensions, and the implementation of the algorithm on the GPU. GPU implementation on a personal computer's graphics card allows a widespread user base to utilize our new LD 3D TFM code without the need to resort to powerful computational cores. The motivation of applying finite or LD continuum mechanics theory in analyzing cellular traction fields was warranted through the experimental observation of existing significant displacement gradient profiles.

To illustrate the significance of the displacement gradient and its role in determining the most appropriate theoretical TFM framework, we provide a quantitative side-by-side comparison of the traditionally SD 3D TFM framework to our LD 3D TFM approach. As shown in Figs. 2–7, the deviation in the recovered surface traction is significant at values of 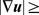
0.5, exceeding 30% error in the determined maximum traction values. Therefore we recommend to always compute the displacement gradient at every time point, to assess the appropriateness of a SD 3D TFM versus a LD 3D TFM mathematical framework. We also show that for quantities such as the total cell force and the strain energy, the deviation errors between the two approaches are small, and a linear framework might provide adequate results. Lastly, we present a simple forward formulation of calculating LD 3D cell surface tractions. Our entire LD 3D TFM algorithm is freely accessible via download from our website (http://franck.engin.brown.edu).

In conclusion, our new LD 3D TFM algorithm presented here provides unprecedented spatial detail of 3D cellular traction fields at computation times of a few minutes on an average personal computer equipped with a GPU graphics card. We show that, depending on the cell-induced displacement gradient magnitude, the traditional linear TFM frameworks such as the Boussinesq theory can introduce significant error (Figs. 2–7) in estimating cellular tractions, and a large deformation formulation should be used instead. To this end, we provide a straightforward method to account for finite deformations, and thereby to accurately calculate cell tractions in any material for which the constitutive material behavior is known.

## Materials and Methods

### Large versus Small Material Deformations

In continuum mechanics, a material point undergoing a deformation from location **x** to location **y** can be represented by its material displacement, 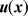 by
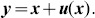
(2)Differentiating both sides with respect to 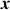 yields
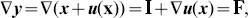
(3)where 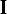 is the identity matrix and 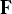 is the material deformation gradient tensor. The gradient tensor physically describes the shearing and stretching motion that a small line segment 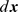 undergoes as it moves from configuration 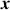 to configuration 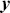. To calculate the material strains associated with that deformation, we compute the Lagrangian strain tensor as
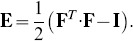
(4)
Equation 4 can be rewritten in terms of the material displacement as
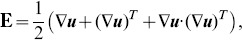
(5)where the term 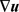 is known as the displacement gradient. Equations 2–5 are valid for any non-linear finite (large) homogeneous deformation. In small strain and linear elasticity, which is the fundamental framework of almost all TFM methodologies, the displacement gradient term is assumed to be small, i.e., 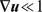, which simplifies Eq. 4 to
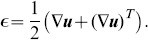
(6)The quantity 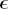 is known as the infinitesimal strain tensor. Most of the previous TFM studies justified this assumption by showing that the measured cellular strains were within 10% of their nominal or principal values [26]–[28]. While several numerical papers have suggested accuracy improvements in calculating cell tractions by using a finite deformation formulation, no prior experimental observation had been made to corroborate the necessity of such approaches.

The observation presented in this paper, that cells can indeed generate non-linear material strains, should not only have implication for traditional TFM methodologies, but also for cell traction assays that utilize very compliant micropillars [29]–[31]. Most micropillar systems calculate the applied post tractions according to the Euler-Bernoulli beam theory, which applies only to small deformations (SD) and strains. This may be entirely adequate if the strains are indeed small, however in the presence of large strains may warrant additional large deformation (LD) analysis.

### LD 3D TFM

**Figure 8:**
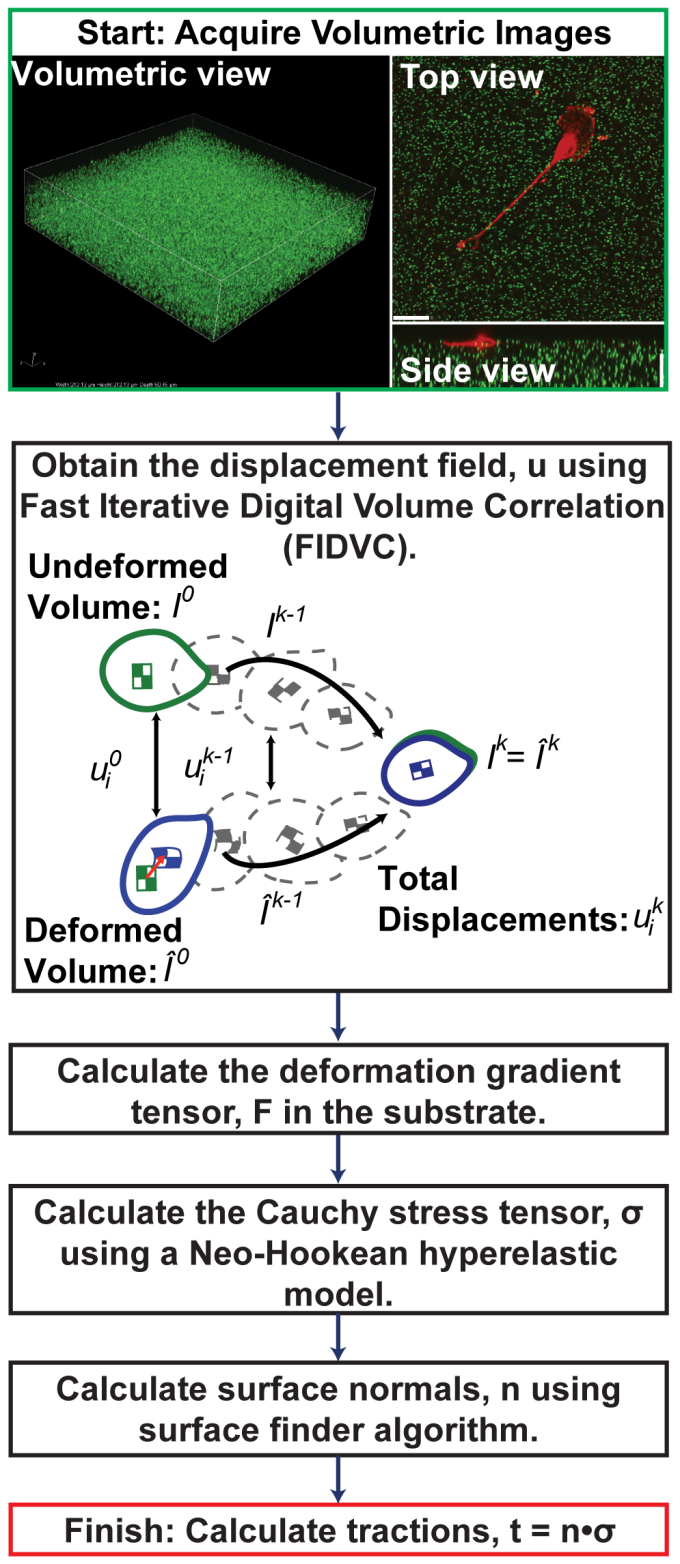
Flowchart of the large deformation high-resolution 3D TFM technique illustrating how cell surface tractions are being calculated.

The two significant advances in improving our traction resolution capability are: (a) a completely new approach to measuring the cell-imposed displacement fields, via our recently developed Fast Iterative Digital Volume Correlation (FIDVC) algorithm [20], and (b) a LD continuum mechanics formulation to determine more accurate cell surface tractions. Figure 8 illustrates the flow of our new LD 3D TFM technique. We will address each part of Fig. 8 in detail below.

Analogous to our previous 3D TFM technique, 3D volumetric image stacks are acquired using laser scanning confocal microscopy (LSCM). The overall dimensions of each stack are user-dependent, but are generally within the range of 512
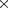
512
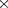
P voxels (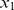 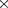 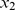 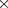 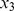), where P ranges from 64 to 256 pixels. Using a resonant Galvano mirror and Piezo objective nosepiece, 3D volumes can be acquired within time lapse intervals as small as ten seconds. To estimate the cell-generated displacements, the motions of embedded submicron-diameter fluorescent particles are estimated using our FIDVC algorithm [20]. The top image in Fig. 8 shows an example of a three-dimensional volumetric image recorded using LSCM, denoting the starting point for our calculations. The submicron particles are shown in green and the cell is superimposed in red.

#### Estimating 3D LD Cellular Displacements

Motivated by the Gaussian-like material displacement field signature that cells generate [6], [10], [12], [13], [32], we developed a new volumetric displacement finding scheme, particularly suited to handle cellular deformation fields. While our previous DVC approach provided a robust approach in estimating cellular displacement fields, it suffered from both slow computation times and lack of high spatial resolution close to the origin of traction application. Capturing highly localized displacement gradients such as those found near the peak traction locust is challenging with any cross-correlation metric, due to the intrinsic low-pass filtering characteristics of such a technique. However, their numerical robustness, high precision and easy implementability makes them the preferred choice over single particle tracking algorithms. To address the challenges of achieving high spatial resolution at low computational costs, we recently developed a new technique called the FIDVC approach [20].

While the mathematical and implementation details of this new FIDVC algorithm can be found elsewhere [20], we provide a brief summary of its key technical points. LD motion estimates are generally captured by a 12 degree of freedom minimization formulation, that accounts for rigid body as well as affine transformation between small regions, or subsets, within an image pair [33]. Such approaches have been well-documented in the DIC communities, but they generally perform poorly in DVC measurements, due to the large computational cost associated with the third dimension [34]. To solve the issue of remaining computationally efficient while computing large deformations in 3D volumetric data sets, we extended the well-known iterative deformation method (IDM) into three dimensions and applied it to calculating volumetric displacement fields. The working principle of the IDM is schematically shown in the second step in Fig. 8, where the deformation field between two volumetric images, 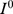 and 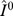, is linearized into 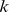-increments by means of incrementally warping both image pairs by estimates of the cumulative displacement field, 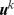. As Fig. 8 shows, the iterative process starts by estimating the displacement field, 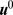, between the two volumetric images, using our previously published DVC cross-correlation formulation [12]. During the next step (k = 1), the cumulative displacement field estimate,
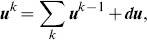
(7)is used to symmetrically warp both the undeformed and deformed images, 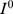 and 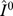, into a new configuration, 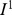 and 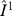, by
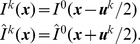
(8)For the first warping operation, i.e., 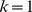, 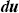 = 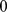. For each linearized k-increment, the DVC cross-correlation algorithm from our previous 3D TFM method is used to calculate the material displacement fields, 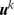, with high accuracy. This process is repeated until both the reference and deformed image have converged to the same intensity pattern, producing the cumulative displacement field measure, 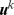. Convergence is generally achieved within less than seven iterations, i.e., 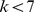.

One of the key benefits of the IDM method is the spatial refinement of the local interrogation window size. In our previous SD 3D TFM method we used a fixed interrogation, or subset, size of 64 voxels, similar to many correlation-based algorithms. This particular choice in subset size is typically governed by the maximum intensity information that can be obtained within the subset, such as the fiducial marker size, density and distribution. Higher particle densities allow for smaller interrogation windows to be used. To provide maximum spatial resolution, the smallest possible subset size is sought while still maintaining high cross-correlation robustness, which is often found at a window size of 64 voxels. However, by employing an interrogation window refinement during the iterative IDM process, our FIDVC technique is capable of reducing its subset size to 32 voxels and in some instances even lower, without introducing significant correlation error. The result provides higher overall spatial resolution than previously possible, which is exemplified in Fig. 1.

#### Estimating 3D LD Cellular Tractions

After the cellular displacements, 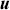, are determined, the deformation gradient tensor, 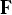, is calculated via finite differences. As shown in Eq. 3, the displacement gradient, 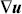, and the deformation gradient tensor, 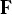, are directly related to the gradients of the measured displacement field. In order to provide the most robust measure of the displacement gradient, we evaluated several differentiation kernels specifically designed to mitigate sampling de-aliasing errors. The current differentiation kernel featured in the here-presented LD 3D TFM technique employs an optimal-11 tab filter, which is described in detail by Farid et al. [35].

Similar to our SD 3D TFM approach, calculating cellular surface tractions first requires determination of the material's true stress tensor, or Cauchy stress 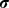. Given that for the LD, the reference and deformed configuration are no longer identical, computation of the Cauchy stress tensor involves the deformation gradient tensor. While our technique is general enough to allow numerical implementation of any constitutive material law, here we present the formulation of the stresses in polyacrylamide (PA), which can be well approximated by a Neo-Hookean material model [22]. Thus its Cauchy stress, 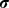, can be expressed [5] as
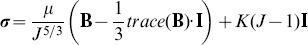
(9)where the parameters 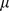, and 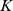 are the material's shear and bulk modulus. Both of these quantities can be related to the Young's modulus of a material by
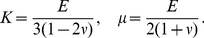
(10)The quantities, 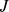, and 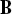 are the Jacobian of 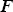, and the left Cauchy Green's tensor, respectively. They are mathematically defined as
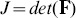
(11)and
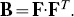
(12)


**Figure 9:**
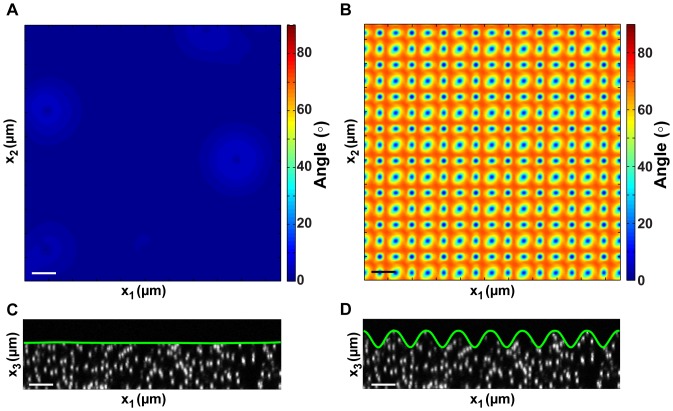
Analytical benchmark validation examples of the free surface finder algorithm. (A) shows the results of the surface finder given a perfectly flat surface, whereas in (B) the surface has regular imposed sinusoidal surface undulations. Scale bars = 20 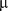
m.

Finally, the surface tractions are calculated via the Cauchy relations, i.e.,
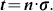
(13)Before Eq. 13 can be evaluated, the surface normals, 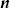, need to be determined. Although it is theoretically possible to estimate the surface normals in the deformed state, via the deformation gradient tensor and the reference surface normals, this procedure is practically challenging, since the reference normals are often not known. To address this issue, we developed a surface finding algorithm that uses the LSCM images to determine the true surface plane, i.e., 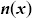. Here, each deformed surface is determined by utilizing the spatial locations of the fluorescent beads in the raw LSCM images. A surface is built from scattered grid data that is calculated using the local maxima of sliding windows along the 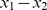 plane. The final surface is generated by a least-squares fitting procedure of the scattered data, ensuring the surface gradient is as smooth as possible [8]. The surface normals, 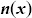, are calculated using the Delauney triangulation representation of the surface. Figure 9 shows two validation test cases of the surface finding algorithm for two surfaces that are numerically simulated from experimentally acquired volumes. In the first example, Fig. 9(A) shows the angle deviation between the calculated surface normal and the analytically imposed surface normal, 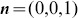. The corresponding cross section of the volumetric image is shown in Fig. 9(C) with the superimposed outline (green) that is found by the surface finding algorithm. Similar validation was performed on a surface that is defined by
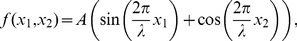
(14)where 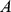 is the amplitude and 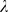 is the wavelength of the surface profile, which were set to 3.6 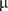
m and 13 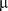
m. The root mean squared difference between the calculated and analytically exact values of the surface wave is within 1%. Using this procedure, the full-field surface normals for each volumetric image are calculated, and the surface tractions along that particular plane are computed according to Eq. 13.

#### Resolution and Measurement Sensitivity

Since the determination of the surface tractions involves calculations of strains and experimental determination of material constants, the sensitivity of our LD 3D TFM technique in terms of traction forces needed to be assessed. This was accomplished by converting the measured displacement and strain fields of control samples (without cells) into surface tractions. Using standard statistical error analysis, we determined the noise floor to be similar to that for our SD 3D TFM technique. Specifically, we can accurately resolve any displacements and strains greater than 0.5 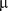
m and 1.0%, respectively, and hence by means of Eqs. 9–13, any stresses and traction forces that are greater than 50 Pa or 50 pN/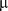
m^2^ for all samples with a Young's modulus of 1.70 kPa. A similar analysis of the maximum resolution sensitivity for 2D and 3D TFM methodologies can be found elsewhere [12], [32], [38].

#### Global Force and Moment Balance

Analogous to our SD 3D TFM methodology, we compute the sum of all forces and moments acting on any given control volume inside each PA gel, to verify that static force and moment equilibrium are satisfied. The overall procedure is identical to our previous methodology [12]. Under all experimental conditions and imaging time points, we found static equilibrium satisfied with force and moment magnitudes on the order of 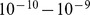 N, and 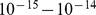 N

m, respectively, which is consistent with our determined experimental noise floor, and similar to our previously reported values [12].

### Materials Preparation

The remainder of the Materials and Methods section is dedicated to briefly describing the experimental conditions under which cell-displacement data was acquired. Most of the described materials procedures are based on our previously described methods and protocols [11], [12], [39], [40].

### Glass Coverslips and Microscope Slides Surface Modification

Circular coverslips (25 mm, Thermo Fisher Scientific, MA) are chemically modified to allow covalent attachment of polyacrylamide substrates, using previously described protocols [2], [11], [41]. Briefly, glass coverslips are rinsed with ethanol and placed in a petri dish containing a solution of 0.5% (v/v) 3-aminopropyltrimethoxysilane (Sigma-Aldrich, MO) in ethanol for 5 minutes. Next, coverslips are washed with ethanol, and submersed in a solution of 0.5% glutaraldehyde (Polysciences, Inc., PA) in deionized (DI) water for 30 minutes. Activated coverslips are washed with DI water and left to dry. Rectangular coverslides (75
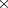
25 mm, Fisher Scientific) are chemically modified to create hydrophobic surfaces, to facilitate gel detachment. Coverslides are placed in a petri dish containing 97% (v/v) hexane (Thermo Fisher Scientific, MA), 2.5% (v/v) (tridecafluoro-1,1,2,2-tetrahydrooctyl)-triethoxysilane (SIT) (Gelest Inc., PA), and 0.5% (v/v) acetic acid (Sigma-Aldrich, MO) for approximately 1 minute. Coverslides are removed and left to dry.

### Preparation and Mechanical Characterization of PA Substrates

Thin films of different PA gels are prepared from 40% w/v acrylamide (Bio-Rad, CA) and 2% w/v N,N-methylene-bis-acrylamide (BIS) (Bio-Rad, CA) stock solutions as described previously [2], [11], [41]. The final acrylamide-BIS concentration ratios yield Young's moduli of 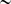
238 Pa for Schwann cells, 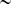
1500 Pa for neutrophils, and 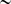
820 Pa for 3T3 fibroblasts. All substrates contain 14% (w/v) of fluorescent microspheres (0.5 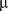
m in diameter, carboxylate-modified, Life Technologies, NY). Crosslinking is initiated through the addition of ammonium persulfate (Sigma-Aldrich, MO) and N,N,N,N-tetramethylethylenediamine (Invitrogen, CA). The PA gel solution is vortexed for about 30 seconds, and 35 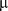
l of PA solution is pipetted on the surface of the microscope slide and sandwiched with an activated glass coverslip (25 mm in diameter), yielding a final thickness of around 60 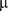
m.

### Mechanical Characterization of PA Substrates

Mechanical characterization of all PA substrates is based on previously established testing protocols on a custom-built uniaxial compression apparatus. Briefly, gel samples are cast in circular nylon molds (16 mm in diameter and 10 mm in height). Following polymerization, the molds are removed and the samples are submersed in DI water. The custom-built compression device consists of a centrally positioned linear actuator (Series A, Ultramotion, NY) and a built-in linear encoder, that provides displacement information with a displacement resolution of 1 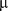
m. To measure the compressive forces acting on each sample, a 50-gram linear force transducer (LCFA-50F, Omega Engineering Inc., CT) is attached to the end of the linear actuator. To ensure uniaxial compression conditions, a ball point tip-platten top is placed on each gel sample, and all samples are carefully aligned along the central compression axis of the linear actuator [11], [21]. Nominal stress (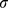) is computed by dividing the measured applied force by the circular contact area. Nominal strain (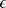) is calculated by measuring the height change of each sample divided by the sample's original height. Each sample is compressed at three different strain rates (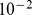, 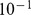, and 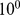) to capture any time-dependence in the material behavior. Young's modulus is calculated from the slope of each linear stress-strain curve. As shown previously, PA can be described as Neo-Hookean solid that has the same Young's modulus as the linear stress-strain relationship in the limit of small strains [22]. The Poisson's ratio is taken to be 0.45, which is within the range of the typical values chosen for TFM studies [26], [42].

### Functionalization of PA Substrates

To promote cell attachment to polyacrylamide films, substrates are functionalized with laminin (Schwann cells) or fibronectin (neutrophils and fibroblasts) using the bifunctional crosslinker, sulfo-SANPAH (Pierce Chemicals, TX) [2], [11], [39]–[41]. Excess water is removed prior to deposition of 100 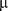
l of sulfo-SANPAH (1 mg/ml) onto the surface of each film, following a 15-minute exposure to UV light. The darkened sulfo-SANPAH solution is aspirated and the procedure is repeated. The samples are thoroughly washed with DI water and are covered with a solution of 0.2 mg/ml laminin (Thermo Fisher Scientific, MA) or 0.2 mg/ml fibronectin (Life Technologies, NY) diluted in 50 mM HEPES (pH 8, Sigma Aldrich, MO). Samples are then left undisturbed at 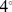
C overnight. Following overnight incubation, the substrates are rinsed three times with 1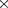 phosphate buffered saline and sterilized with UV irradiation before depositing cells.

### Cell Culture

Schwann cells were cultured as previously described by Mitchel et al. [39]. Fibroblasts and neutrophils were cultured according to protocols published by Franck et al. and Oakes et al. [11], [12], [40]. Schwann cells, fibroblasts and neutrophils were seeded at a density of 1,000, 80,000, and 50,000 cells/cm^2^ on PA gels. Cells were allowed to attach for 4 hours before initiation of the time-lapse microscopy.

### Live Cell Imaging

After an initial seeding period, three-dimensional image stacks of individual cells on PA gels are acquired using a Nikon A-1 confocal system mounted on a TI Eclipse inverted optical microscope, controlled by NI-Elements Nikon Software. Red fluorescent microspheres are used as fiducial markers and excited with a Diode (561 nm) laser. 512
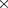
512
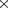
P voxels (209
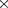
209
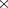
P 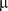
m) confocal volume stacks are recorded at user specific time intervals (e.g., 10 seconds to 30 minutes) with P ranging from 64–256 voxels (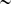
20–77 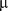
m). To ensure physiological imaging conditions within the imaging chamber, temperature and pH are controlled at 37°C and 7.4 during time-lapse recording, as previously described [11], [12]. Cellular surface outlines are determined either from phase images or from fluorescent membrane labels.

## Supporting Information

Figure S1 **Analytically calculated Gaussian traction dipoles on the substrate surface.** Contour plot of the analytically calculated traction vector magnitude due to the prescribed Gaussian displacement dipoles presented in Fig. 4.
(TIF)

Figure S2
**Measurement sensitivity thresholds in terms of Cauchy stresses and surface tractions as a function of substrate stiffness.** A plot showing the noise floor of (*A*) the Cauchy stress (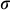) calculated from the deformation gradient that was corrupted with Gaussian white noise with standard deviations of (*red*) 0.1%, (*green*) 1%, and (*blue*) 10%; and (*B*) the tractions are calculated from the Cauchy stress via the Cauchy relation. The [0,0,1] normal vector was additionally corrupted with an appropriate level of Gaussian white noise. To compute the final traction noise floor, the largest standard deviation for each Cauchy stress component was chosen, thus representing the most conservative traction noise floor estimate.(TIF)

Figure S3
**Experimental example of a migrating Schwann cell on the surface of a 3D LSCM imaging volume (Cell Example 2).** (A) Magnitude of the 3D Schwann cell surface displacement field, 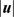, and its (B) resulting displacement gradient magnitude (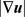). Calculated maximum principal strains from the infinitesimal (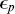) (C), and Lagrangian (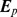) strains (D). The corresponding traction magnitudes calculated on the (E) undeformed surface, 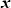, using a linear elastic constitutive model, 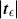, and on the (F) actual deformed surface, 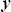 using a large deformation (LD) constitutive model 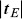. Cell outlines are shown in white. Scale bars = 40 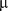
m.(TIF)

Figure S4 **Comparison of commonly reported metrics in TFM for cell example 2.** Side by side comparison of the (A) total force, (B) root mean squared (RMS) tractions and maximum tractions, and (C) strain energy for both the linear elastic, small deformation (SD) and non-linear, large deformation (LD) models.
(TIF)

Figure S5
**Experimental example of a migrating Schwann cell on the surface of a 3D LSCM imaging volume (Cell Example 3).** (A) Magnitude of the 3D Schwann cell surface displacement field, 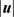, and its (B) resulting displacement gradient magnitude (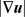). Calculated maximum principal strains from the infinitesimal (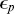) (C), and Lagrangian (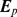) strains (D). The corresponding traction magnitudes calculated on the (E) undeformed surface, 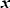, using a linear elastic constitutive model, 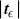, and on the (F) actual deformed surface, 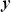 using a large deformation (LD) constitutive model 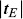. Cell outlines are shown in white. Scale bars = 40 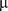
m.(TIF)

Figure S6 **Comparison of commonly reported metrics in TFM for cell example 3.** Side by side comparison of the (A) total force, (B) root mean squared (RMS) tractions and maximum tractions, and (C) strain energy for both the linear elastic, small deformation (SD) and non-linear, large deformation (LD) models.
(TIF)
