# Supplementary figures and images for: High Resolution, Large Deformation 3D Traction Force Microscopy
Source: PLoS One. 2014 Apr 16;9(4):e90976. doi: 10.1371/journal.pone.0090976 (PMC3989172; doi:10.1371/journal.pone.0090976)

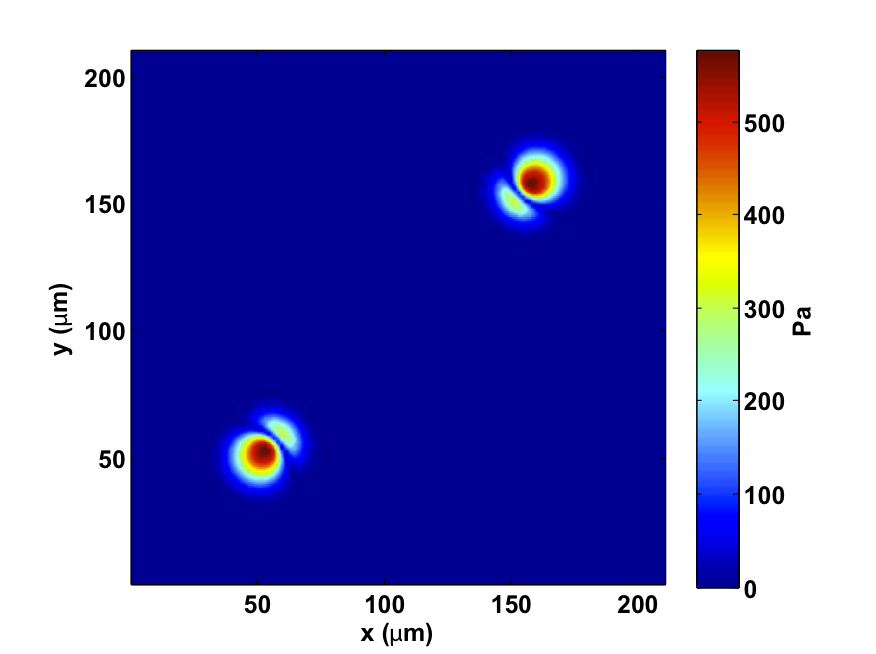

Supplement: Figure S1 — Analytically calculated Gaussian traction dipoles on the substrate surface. Contour plot of the analytically calculated traction vector magnitude due to the prescribed Gaussian displacement dipoles presented in Fig. 4. (TIF) [file pone.0090976.s001.tif]

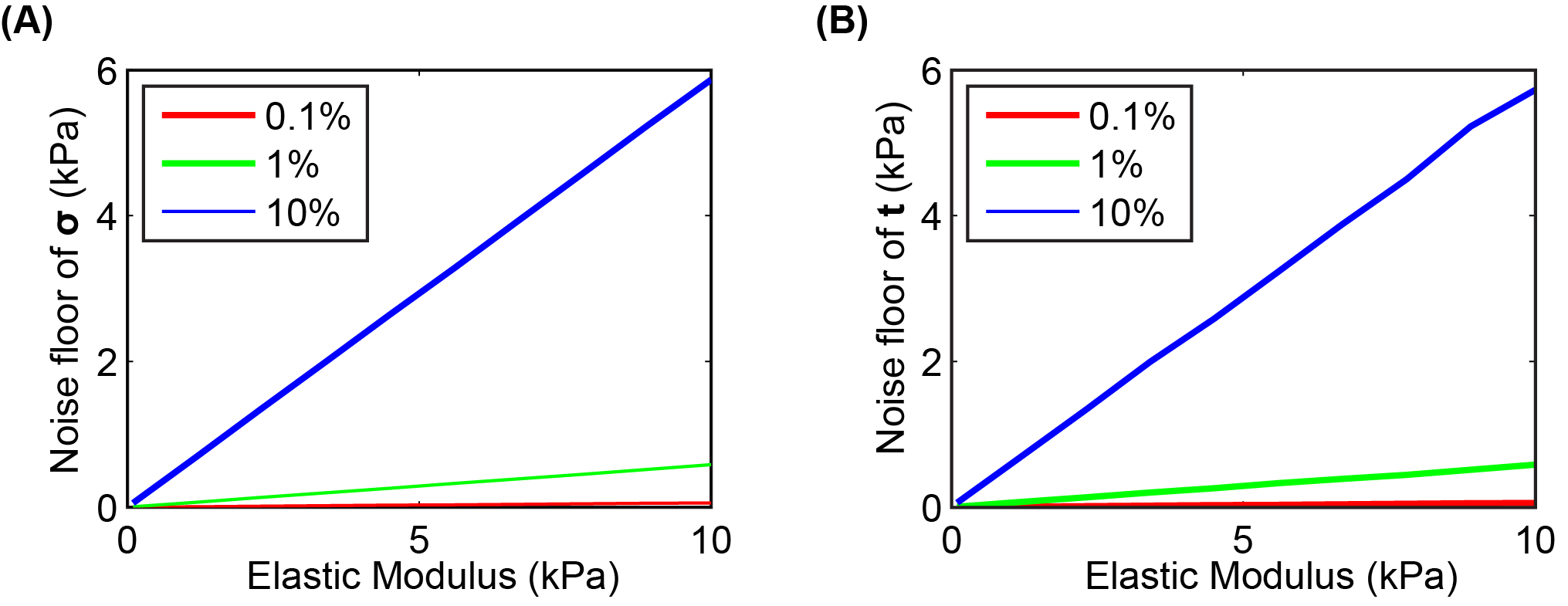

Supplement: Figure S2 — Measurement sensitivity thresholds in terms of Cauchy stresses and surface tractions as a function of substrate stiffness. A plot showing the noise floor of (A) the Cauchy stress () calculated from the deformation gradient that was corrupted with Gaussian white noise with standard deviations of (red) 0.1%, (green) 1%, and (blue) 10%; and (B) the tractions are calculated from the Cauchy stress via the Cauchy relation. The [0,0,1] normal vector was additionally corrupted with an appropriate level of Gaussian white noise. To compute the final traction noise floor, the largest standard deviation for each Cauchy stress component was chosen, thus representing the most conservative traction noise floor estimate. (TIF) [file pone.0090976.s002.tif]

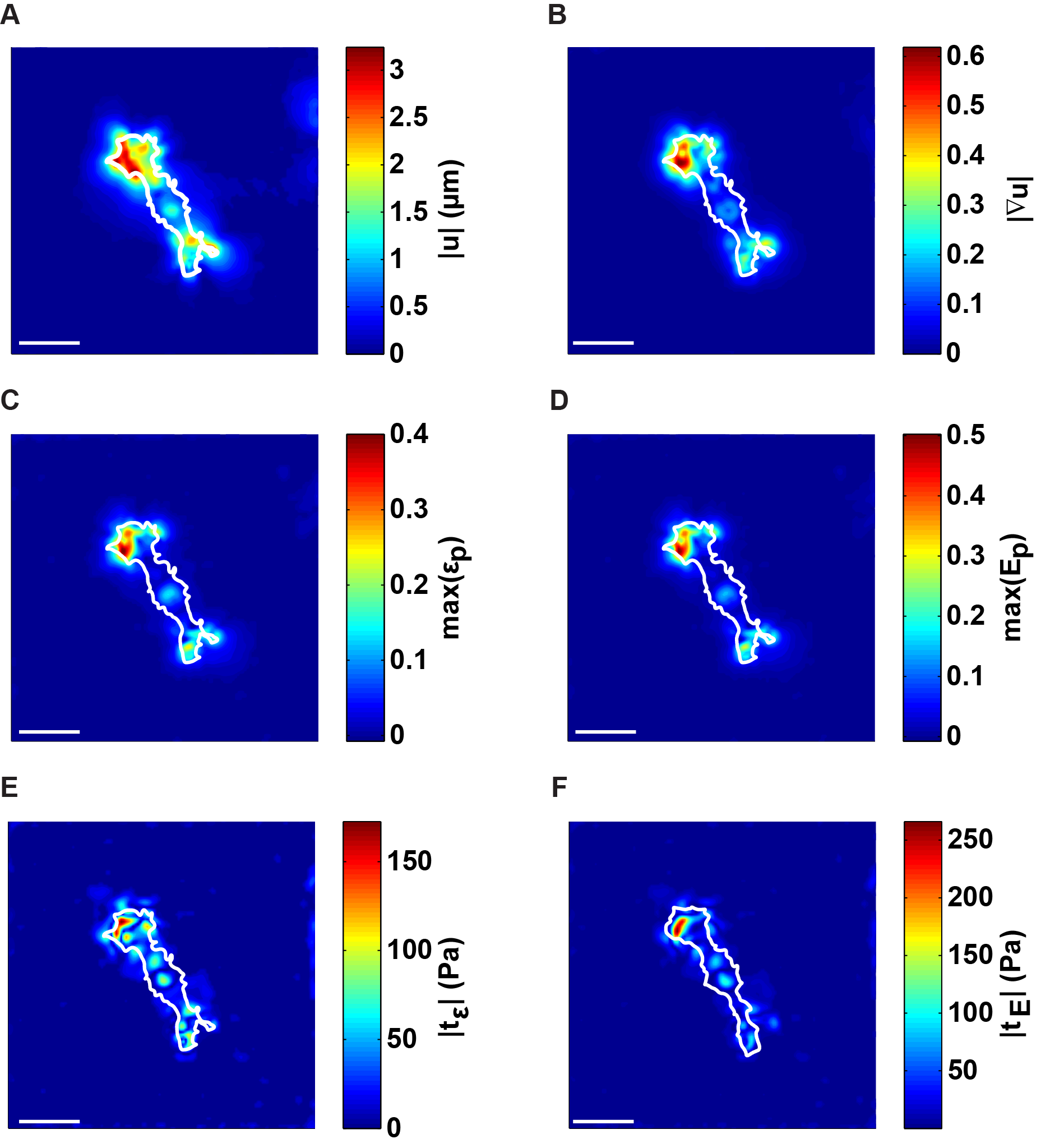

Supplement: Figure S3 — Experimental example of a migrating Schwann cell on the surface of a 3D LSCM imaging volume (Cell Example 2). (A) Magnitude of the 3D Schwann cell surface displacement field, , and its (B) resulting displacement gradient magnitude (). Calculated maximum principal strains from the infinitesimal () (C), and Lagrangian () strains (D). The corresponding traction magnitudes calculated on the (E) undeformed surface, , using a linear elastic constitutive model, , and on the (F) actual deformed surface, using a large deformation (LD) constitutive model . Cell outlines are shown in white. Scale bars = 40 m. (TIF) [file pone.0090976.s003.tif]

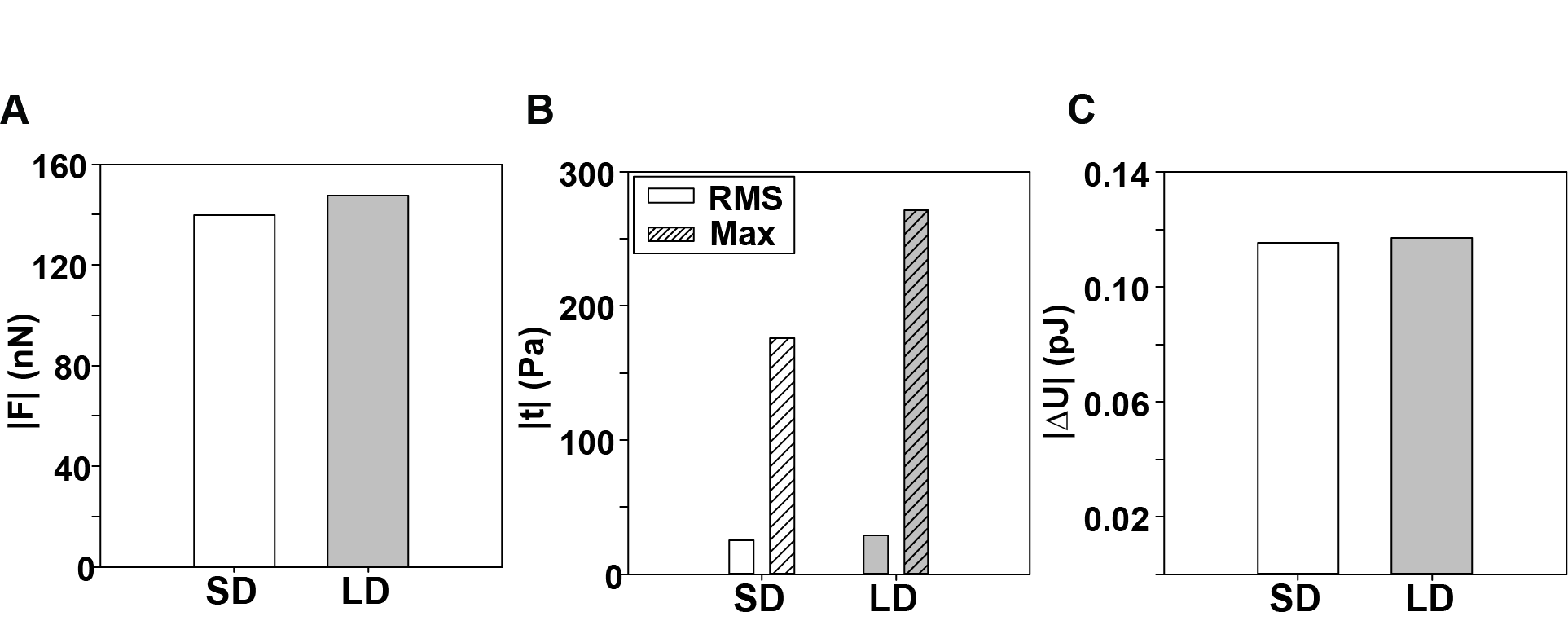

Supplement: Figure S4 — Comparison of commonly reported metrics in TFM for cell example 2. Side by side comparison of the (A) total force, (B) root mean squared (RMS) tractions and maximum tractions, and (C) strain energy for both the linear elastic, small deformation (SD) and non-linear, large deformation (LD) models. (TIF) [file pone.0090976.s004.tif]

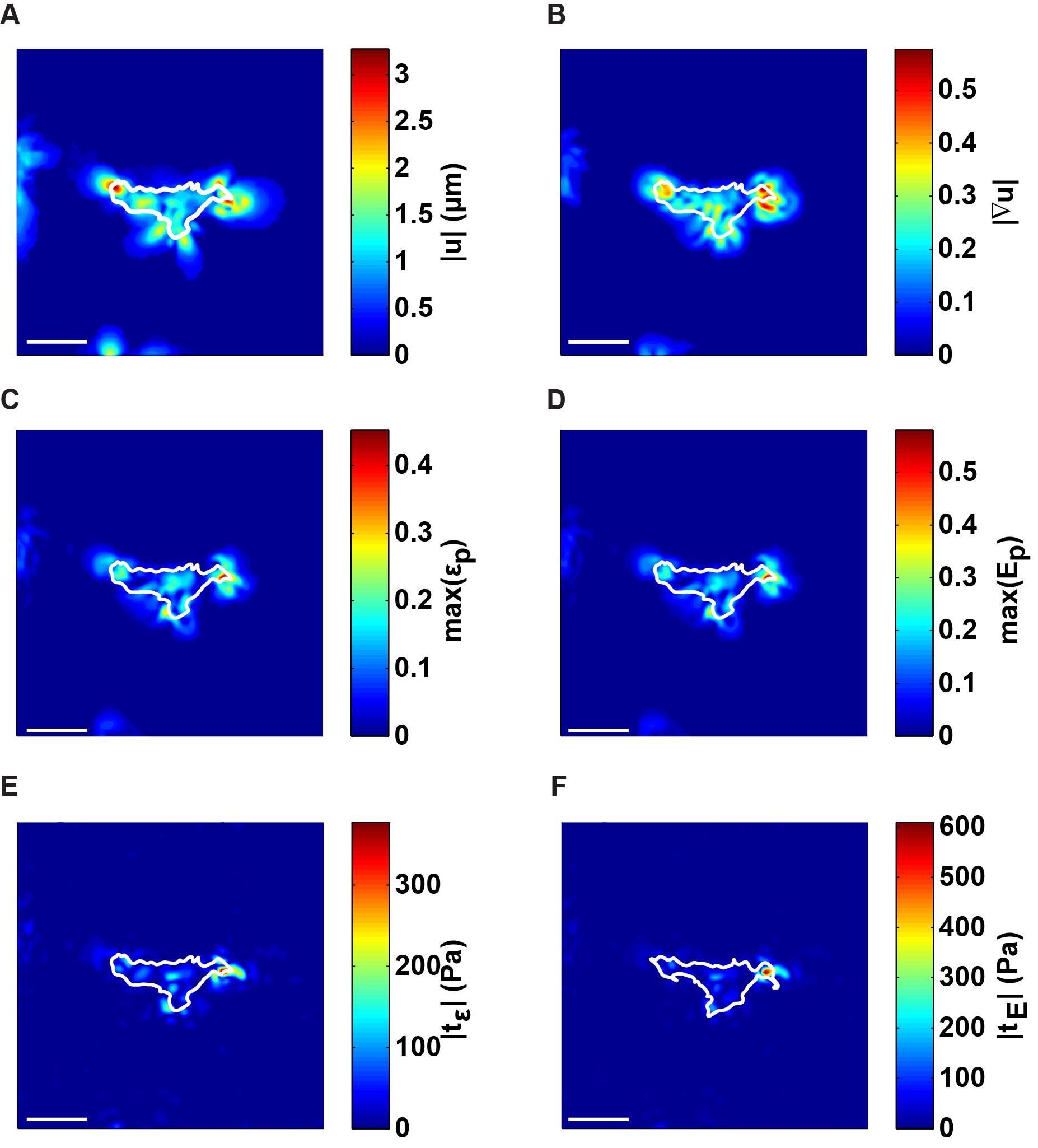

Supplement: Figure S5 — Experimental example of a migrating Schwann cell on the surface of a 3D LSCM imaging volume (Cell Example 3). (A) Magnitude of the 3D Schwann cell surface displacement field, , and its (B) resulting displacement gradient magnitude (). Calculated maximum principal strains from the infinitesimal () (C), and Lagrangian () strains (D). The corresponding traction magnitudes calculated on the (E) undeformed surface, , using a linear elastic constitutive model, , and on the (F) actual deformed surface, using a large deformation (LD) constitutive model . Cell outlines are shown in white. Scale bars = 40 m. (TIF) [file pone.0090976.s005.tif]

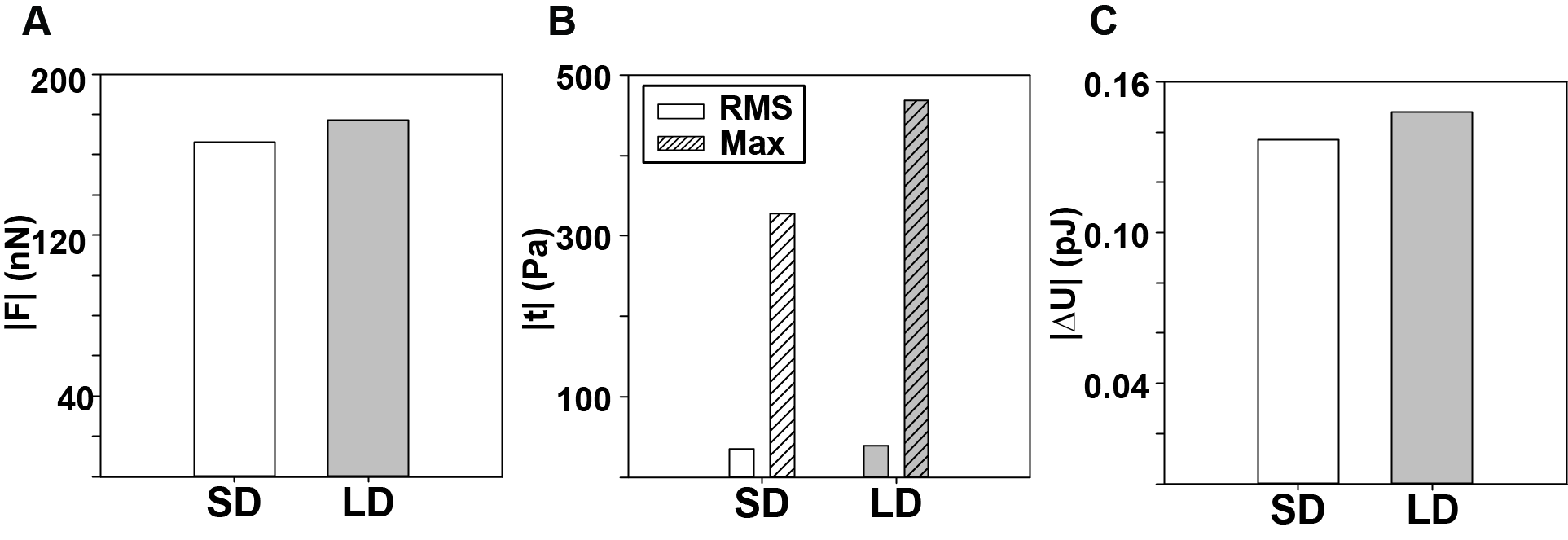

Supplement: Figure S6 — Comparison of commonly reported metrics in TFM for cell example 3. Side by side comparison of the (A) total force, (B) root mean squared (RMS) tractions and maximum tractions, and (C) strain energy for both the linear elastic, small deformation (SD) and non-linear, large deformation (LD) models. (TIF) [file pone.0090976.s006.tif]
